# Global, regional, and national prevalence and determinants of volunteer work: a meta-analysis study using survey data

**DOI:** 10.3389/fsoc.2025.1584761

**Published:** 2025-07-07

**Authors:** Samuel Manda, Nada Abdelatif, Dineo Seabe, Sollie Millard, Tapiwa Kamuruko

**Affiliations:** ^1^Department of Statistics, University of Pretoria, Pretoria, South Africa; ^2^Biostatistics Research Unit, South African Medical Research Council, Cape Town, South Africa; ^3^The Nelson Mandela School of Public Governance, University of Cape Town, Cape Town, South Africa; ^4^Voluntary Advisory Services Section, United Nations Volunteers (UNV), Bonn, Germany

**Keywords:** volunteering, meta-analysis, non-profit, SDGs, data sources (DS)

## Abstract

**Introduction:**

While there is an increasing recognition of the role of volunteer work in promoting global development, the factors influencing volunteering at both global and regional levels remain poorly understood. This study employed a random-effects meta-analysis to estimate the prevalence of volunteering and to investigate variations in volunteer rates by gender, educational level, and age, both globally and at regional and country levels.

**Methods:**

We analyzed data from 49,458 volunteers aged 15 and older across 37 World Values Surveys (WVS) conducted between 2000 and 2018 in 31 countries. Random-effects meta-analysis was employed to calculate the overall prevalence of volunteering and to compare volunteer rates among different demographic groups: females vs. males, individuals with at least a secondary education vs. those with primary education or less, and individuals aged 35 years or older versus those under 35 years.

**Results:**

The overall pooled prevalence of volunteering was found to be 39.93% (95% CI: 33.25–46.62). Significant disparities in volunteering rates were observed across regions and countries, with the highest rates recorded in Africa (61.15%, CI: 50.54–77.77) and the lowest in Europe (28.97%, CI: 19.43–38.51). Rates varied considerably, from a low of 19.16% in Russia (CI: 19.16%–22.76%) to a high of 80.27% in Tanzania (CI: 77.99–82.55%) and 72.00% in Uganda (CI: 69.18–76.22%). Volunteering in religious organizations was the most common type, accounting for 16.77% (CI: 13.76–19.78), followed by community and health organizations at 14.62% (CI: 11.74–17.50). Regional differences were notable, with the highest rates of religious volunteering in Africa at 41.09% (CI: 20.17–62.02) and the lowest in Europe at 8.10% (CI: 5.25–10.95). The pooled relative risks for gender, educational, and age differentials were (RR = 0.91, 95% CI: 0.86–0.97); (1.20, CI: 1.18–1.36); and (1.00, CI: 0.95–1.05), respectively, indicating that only educational differentials significantly affected volunteering rates. The impact of education on volunteering was more pronounced in Europe (1.54, CI: 1.21–1.97) compared to Africa (1.17, CI: 1.03–1.33).

**Conclusions:**

Volunteering rates vary significantly by region and country, often correlating with individuals' educational levels. These findings are essential for policymakers aiming to enhance volunteer initiatives. By understanding the regional contexts and factors, such as the influence of education level on volunteering, policymakers can develop tailored programs that attract new volunteers and promote retention, ultimately fostering greater community engagement and social cohesion.

## Introduction

Volunteers strengthen community relationships and trust, advocate for policy changes to support marginalized and underserved populations, and foster cooperation and innovation (International Labour Organization, 2021; United Nations Volunteers, 2021a,b). Through their efforts, many challenges, including poverty, hunger, health issues, inequality, and the need for inclusive, safe human settlements, are addressed, particularly in countries in the Global South (Russell, 2016; International Labour Organization, 2021; United Nations Volunteers, 2021a,b). Researchers and various stakeholders increasingly recognize that, just as volunteering contributed to the Millennium Development Goals (MDGs), it is also essential for achieving many countries' Sustainable Development Goals (SDGs) (Haddock and Devereux, 2016; Russell, 2016; Allum and Devereux, 2020; Plan of Action, 2020a,b). As many as over 860 million volunteers worldwide engaged in various roles through informal and organizational-based volunteering (Salamon et al., 2018; United Nations Volunteers, 2021b), there is a growing appreciation for volunteerism's unique contributions to addressing social, economic, and environmental challenges at local, national, and global levels. However, data on the scale and scope of volunteering and the factors that influence it still need to be made available.

Understanding and evaluating the individual factors associated with volunteering can help governments and policymakers develop programs and encouragements to attract potential volunteers, ultimately supporting communities more effectively (Seabe, 2014; Anheier and Salamon, 1999). However, an empirical assessment of the scope of volunteerism and its determinants is limited due to the unavailability of volunteer work data. Volunteer work is often part of national labor force surveys in developed countries. Developing countries have yet to measure volunteering consistently (Yimer, 2020; Logan et al., 2020). One program that has attempted to collect volunteer work data with an extensive geographical scope using standardized modules and questionnaires is the World Values Survey (WVS) (https://www.worldvaluessurvey.org).

This paper presents summary statistics on the prevalence of volunteering and its associations with age, gender, and education, derived from 37 WVS datasets collected across 31 countries. It offers valuable insights at both global and continental levels while also highlighting variances in volunteering practices. This work contributes innovative perspectives by applying existing data on volunteering to the 2,030 Agenda and the Sustainable Development Goals (Plan of Action, 2020b; United Nations Volunteers, 2021a,b). Prior systematic reviews and meta-analyses concerning volunteering have significantly advanced our understanding of factors influencing volunteerism. However, many of these studies primarily concentrated on the impact of formal volunteering on volunteers' health and wellbeing (Jenkinson et al., 2013; Okun et al., 2013; de Wit et al., 2022; Nichol et al., 2024), motivations and satisfaction among volunteers (Zhou and Muscente, 2023), volunteer turnover (Forner et al., 2024), student volunteers in health-related contexts (Mahsusi et al., 2024), or volunteering among older populations (Okun et al., 2013; de Wit et al., 2022). In addition to being population-specific, these reviews often combined findings from studies employing varied designs and methodologies. Our objective was to address these limitations by conducting a two-stage meta-analysis, wherein the first stage involves estimating prevalence and associations from individual datasets collected using standardized measurement and collection tools. Consequently, our findings aim to bolster confidence in the conclusions derived from the results.

Multiple demographic factors, such as gender, age, and race, significantly affect an individual's likelihood of volunteering and indirectly influence other key determinants of this behavior (Wilson and Musick, 1997). Research consistently shows that gender differences play a significant role, with women volunteering more than men. This may stem from societal norms and gender role stereotypes (Taniguchi, 2006; Seabe, 2014). Regarding age effects, motivation to volunteer tends to evolve and shift across different age groups, with younger volunteers often driven by the acquisition of career skills, experience, and personal development. Meanwhile, older adults may prioritize more meaningful social engagement and making an impact. These changing priorities have been explained as resulting from life course factors, such as family formation, career considerations, and transitions from the paid workforce to retirement, health changes, widowhood, and reductions in social network size (Dávila and Díaz-Morales, 2009; Butrica et al., 2009; Hank and Erlinghagen, 2010). Generally, empirical research has suggested that the association between volunteering participation rates and age has both a negative linear and a negative quadratic relationship, indicating a curvilinear trajectory in age effects (Choi et al., 2007; Einolf and Chambre, 2011; Han et al., 2023). However, in a few instances, the relationship between age and volunteering is linear, with increasing participation rates in some societies (Seabe, 2014; Fondling et al., 2023; Logan et al., 2020). Younger volunteers are usually driven by career advancement and personal development, whereas older volunteers often focus more on social concerns than the desire to make new friends.

Regarding race, in countries where race is closely associated with socioeconomic status and culture, there has been conflicting evidence. In South Africa, the black population volunteers more than their white counterparts (Seabe, 2014; Fondling et al., 2023). In the United States, non-white individuals have been found to have lower rates of volunteering (Fondling et al., 2023; Han et al., 2023). Due to confounding issues related to race as a predictor and differences in racial composition between countries, this analysis will not consider race. The impact of demographic factors on volunteering is complex. It is influenced by other elements such as human capital (including education, income, and wealth), social capital (such as social relationships and membership in associations), health status (overall health and disability), and cultural capital (such as religiosity) (Logan et al., 2020; Han et al., 2023; Seabe, 2014) thoroughly discussed the various individual factors influencing individual volunteering. Regarding contextual factors affecting individual likelihood of volunteering, Enjolras (2021) discusses several of them, including economic, political, social, and religious contexts. This paper explores the extent and nature of volunteer work, considering age, education, and gender, to evaluate variations in volunteering behavior.

Research studies have shown significant differences in volunteering rates across various countries, regions, and continents. For example, Gesthuizen and Scheepers (2012) and Enjolras (2021) used quantitative multilevel models to identify substantial variations in formal volunteering among 17 countries studied by the Organization for Economic Cooperation and Development (OECD) and 23 European countries, respectively. These differences were attributed to country-level wealth, income inequality, political tolerance, and social and religious diversity. On the other hand, Logan et al. (2020) analyzed civic engagement data from Afrobarometer surveys across 37 African countries. They found that wealthier countries tend to report lower levels of volunteerism, while democracies generally report higher levels. Salamon et al. (2018) provided a more in-depth analysis of volunteering rates, highlighting significant variations across different regions. This variability was explained by macro-level factors influencing individuals' capacity to volunteer, including economic, human, political, social, and religious contexts. Differences observed between countries may also arise from the appropriateness of local volunteering measurements and specific volunteering behaviors (Russell, 2016). Through meta-analysis in cross-border studies, Allik and Realo (2004) found that in countries with high GDP, a long history of political systems, and Protestants as the majority, residents participate in volunteering activities more frequently. On the other hand, Aydinli et al. (2016) found that cultural differences between societies and countries play a complex role in motivation to volunteer. Changes in the community and community-related variables, including socio-cultural value (individualism and collectivism), socio-demographic and socio-economic features, or political characteristics, impacted the scale and scope of volunteering (Aydinli et al., 2013).

To our knowledge, no study has comprehensively measured the scale and scope of volunteer work and how it correlates with differences in gender, age, and educational level on a global scale. This study aims to fill this gap by conducting a meta-analysis of volunteering prevalence using multiple datasets from the World Values Survey program, which employs consistent tools and methodologies for data collection across countries. Our method will enhance the objectivity and generalizability of our findings while increasing the statistical power of our analysis. This research will provide valuable insights into volunteerism's overall reach and impact, an area that warrants further understanding. Additionally, the findings will support the United Nations Volunteers (UNV) initiative to assess the scale and scope of volunteer efforts using available data.

## Methods

### Data

The study used volunteering prevalence data reported by over 49,458 persons aged at least 15 years in 37 World Values Surveys (WVS) conducted between 2000 and 2018 across 31 countries worldwide (Inglehart et al., 2014). The World Values Survey (WVS) (http://www.worldvaluessurvey.org) is an international research programme of social scientists and researchers that provide nationally representative household surveys that provide data on people's social, political, economic, religious, and cultural values worldwide. Eight successive waves have been completed across over 120 societies on all six continents, representing 94.5% of the world's population.

### Measures of volunteering in World Values Surveys (WVS) programme

The World Values Survey (WVS) data sets include demographic and socioeconomic variables, as well as critical subjective questions about whether the sampled individuals engaged in unpaid voluntary work for any of six types of organizations: religious groups, sports, women's, professional and political groups, community health, and others. This engagement was evaluated using a set of 14 questions. Our study defined overall volunteering as any indication of unpaid work in any organization, as Seabe (2014) described.

### Statistical analysis

Random effects meta-analyses were implemented to produce global and continental estimates of the prevalence of volunteering and its association with age, education, and gender. Results are presented using forest plots that show the pooled prevalence and association in each region and period, along with their 95% confidence intervals (CIs) for each study. Heterogeneity between reported prevalence rates was assessed by conducting the Chi-square test, Q-statistics, and I^2^ test (Higgins et al., 2003). Based on the statistical test results, if significant heterogeneity is observed among the included studies, a random-effects meta-analysis model would be conducted to estimate overall pooled effects worldwide and within the five continents. The reference category for gender was male, while the reference categories for age and education levels were individuals under 35 years old and those with primary education or less, compared to secondary education, respectively, when estimating relative risks. The 35 cut-off for age is based on the work of Newman and Newman (2014), among others, who defined four life stages: late adolescence (18–24), early adulthood (25–34), middle (35–60), and late adulthood (61–75). Therefore, the age of 35 could be considered the midpoint between adolescence and early adulthood, as well as middle and late adulthood.

Results are presented using forest plots that display point prevalence and relative risk estimates, along with 95% confidence intervals, for each survey dataset and the pooled results. Subgroup meta-analyses were performed between continents to investigate the sources of heterogeneity in the meta-analysis findings. All analyses were conducted using Stata 17.0 and the *metan* command.

## Results

**Figure 1:**
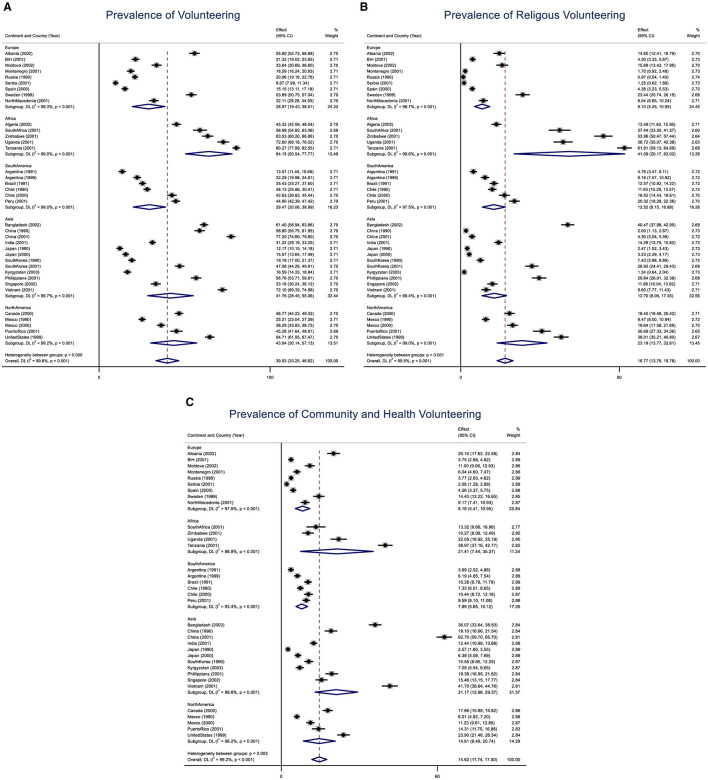
**(A)** Prevalence of any volunteering activity according to the region and country. The dotted vertical line represents the prevalence of the pooled results, with a 95% confidence interval. **(B)** Prevalence of volunteering activity in a religious organization according to the region and country. The dotted vertical line represents the prevalence of the pooled results, with a 95% confidence interval. **(C)** Prevalence of volunteering activity in community and health organizations according to the region and country. The dotted vertical line represents the prevalence of the pooled results, with a 95% confidence interval.

Survey-specific and pooled prevalence estimates of any volunteering are presented in Figure 1A by region and country, with 95% confidence intervals. The dotted vertical line represents the prevalence of the pooled result. The overall volunteering rate was estimated at 39.93% [95% Confidence Interval (CI): 33.25%−46.62%]. However, the included survey data sets exhibited significant heterogeneity in volunteering rates (*I*^2^ = 99.6, *p* < 0.001), ranging from 19.16% (19.16%−22.76%) in Russia to 80.27% (CI: 77.99%−82.55%) in Tanzania, with Uganda reporting a rate of 72.00% (CI: 69.18%−76.22%). Continental results showed that the highest pooled estimates of volunteering were in Africa (61.15%; 50.54%−77.77%), followed by North America (43.64%; 30.14%−46.62%). At 16.77% (13.76), volunteering in religious organizations was the most preferred type of volunteering, followed by volunteering in community and health organizations, which had a rate of 14.62% (11.74–17.50) (Figures 1B, C). Continental variations in religious volunteering were notable, with the highest rates observed in Africa at 41.09% (20.17–62.02) and the lowest in Europe at 8.10% (5.25–10.95). Similarly, the rates for volunteering in community and health organizations varied significantly by continent. Africa and Asia had the highest community and health volunteering rates at 21.41% (7.44–35.37) and 21.17% (12.96–29.37), respectively, while South America recorded the lowest rate at 7.89% (5.65–10.12).

**Figure 2:**
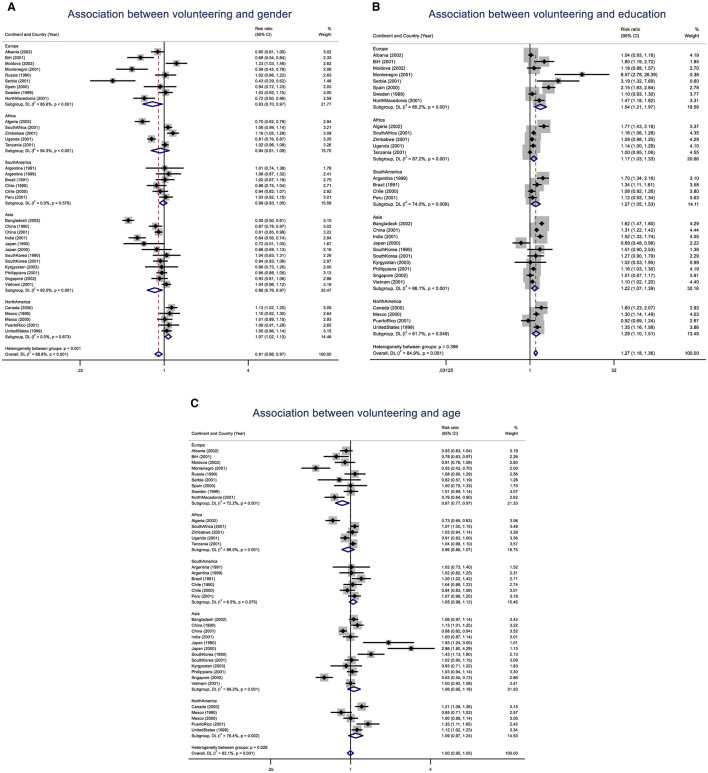
**(A)** The likelihood of volunteering among females compared to males across different regions and countries, accompanied by a 95% confidence interval. The dashed vertical line represents the risk ratio of the pooled results. In contrast, the solid vertical line at the value of 1 indicates no difference in volunteering rates between females and males. **(B)** The likelihood of volunteering among individuals with at least a secondary education, compared to those without or with only a primary education, is presented across different regions and countries alongside a 95% confidence interval. The dashed vertical line indicates the risk ratio of the pooled results. In contrast, the solid vertical line at the value of 1 signifies no difference in volunteering rates between education levels. **(C)** The likelihood of volunteering among individuals aged at least 35 years compared to those aged <35 across different regions and countries, presented alongside a 95% confidence interval. The dashed vertical line indicates the risk ratio of the pooled results. In contrast, the solid vertical line at the value of 1 signifies no difference in volunteering rates between the two age group levels.

Figures 2A–C illustrate the likelihood of volunteering, using three individual-level indicators, namely gender, education and age, presented as relative risk (RR) alongside a 95% confidence interval. The dashed vertical line indicates the risk ratio of the pooled results. The solid vertical line at the value of 1 signifies no difference in volunteering rates between the two groups. It is demonstrated that individual-level factor differences in volunteering exhibit significant variability across countries, continents, and within continents. Females were less likely to undertake volunteer work in many countries in Africa, Asia, and South America. Only in North America did females show a higher likelihood of volunteering than males (RR: 1.07; 1.02–1.13). However, the pooled gender association shows that volunteering was relatively evenly distributed between females and males [Risk Ratio (RR) of 0.91, 95% CI: 0.86–0.97]. Age differences across regions and countries were notable. In Japan, significant disparities were observed, with a risk ratio of 1.93 for older individuals in 1990 (CI: 1.24–3.00). This ratio increased to 2.88 in 2000 (CI: 1.90–4.29). Conversely, older individuals were less likely to volunteer than younger ones in Singapore, with a risk ratio of 0.63 (CI: 0.55–0.73). Similarly, in Montenegro, the risk ratio was estimated to be 0.55 (CI: 0.43–0.70). Regionally, only in Europe was there a significant association between age and volunteer work, with older people being less likely to volunteer (0.87; CI: 0.77–0.97). The pooled estimate of age differences in volunteering was not significant (RR: 1.00; 95% CI: 0.95–1.05), indicating no substantial change in volunteering rates with age globally. Only educational differences in volunteering were significant, with individuals with secondary or higher education having a pooled estimated relative risk of 1.2 (95% CI: 1.18–1.36). This effect was particularly pronounced in Europe, where the risk ratio is 1.54 (95% CI: 1.21–1.97). In contrast, Africa showed the lowest educational effect on volunteering rates, with a relative risk of 1.17 (95% CI: 1.03–1.33). Montenegro had the most significant and most prominent education difference in volunteering [RR: 8.57 (95% CI: 2.78–26.39)], followed by Serbia [RR: 3.19 (95% CI: 1.32–7.69)].

### Sensitivity analysis

There were notable differences in the prevalence of volunteer work and its determinants across the regions and countries examined in the meta-analyses. These differences could have influenced the validity of the pooled estimates and highlighted the potential impact of outliers that might have distorted the overall results. To thoroughly assess the robustness of our meta-analysis findings, we employed a leave-one-out sensitivity analysis method. The results from the 37 meta-analyses conducted with this approach consistently aligned with the pooled estimates. This consistency strengthens our confidence in the robustness of the overall meta-analysis results and addresses concerns about the potential influence of outliers on the findings and conclusions.

## Discussion and conclusions

This study aimed to analyze a more extensive set of volunteering data from 37 World Values Surveys (WVS) datasets collected through standardized methods from individuals aged 15 and older in 31 countries worldwide. This approach enabled us to estimate the global prevalence of volunteering and to examine how factors such as age, gender, and education level influence volunteering rates. Our analysis offers a more comprehensive overview of volunteering worldwide and presents more substantial empirical evidence than similar studies that relied on cross-sectional surveys. Previous research has often focused on specific countries (for example, Seabe, 2014; McGarvey et al., 2019; Fondling et al., 2023; Yimer, 2020) or covered multiple countries (Gesthuizen and Scheepers, 2012; Logan et al., 2020; Enjolras, 2021). While these earlier studies provide valuable insights into the scope and scale of volunteering, they fail to deliver an in-depth global analysis and do not account for variations in individual capabilities related to volunteering. Furthermore, some earlier studies, such as Salamon et al. (2018), utilized data from diverse sources that employed different methodologies. This inconsistency makes it challenging to compare findings across countries and continents.

We found much variation in volunteering rates between countries and continents. The pooled prevalence of volunteering has been estimated at 39.93% [95% Confidence Interval (CI): 33.25%−46.62%], ranging from 19.16% (CI: 19.16%−22.76%) in Russia to 80.27% (CI: 77.99%−82.55%) in Tanzania, with Uganda reporting a rate of 72.00% (CI: 69.18%−76.22%). Continental results have shown that the highest pooled estimates of volunteering were in Africa (61.15%; CI: 50.54%−77.77%), followed by North America (43.64%; CI: 30.14%−46.62%). Similar findings of large and significant variation levels of volunteerism have also been observed across 17 Organizations for Economic Cooperation and Development (OECD) countries using volunteer data from the International Adult Literacy Survey (IALS) (Gesthuizen and Scheepers, 2012) in Enjolras (2021) between 23 European countries using the European Union (EU) Survey on Income and Living and in Logan et al. (2020) between 34 African countries using data from Afrobarometer surveys.

Our findings regarding variations in volunteering between countries and continents are consistent with previous studies, although the explanations for these findings may differ. For example, Enjolras (2021) attributed the observed variations in volunteering to other institutional arrangements across Europe. Countries with low socioeconomic inequality, due to high levels of redistribution, and high social trust have higher volunteering rates than countries with high inequality and low social trust. However, this is also true in some countries, where low inequality coexists with lower levels of social trust. The differences observed in our study could be due to variations in the distribution of resources at the macro level, where enhanced resources allow individuals to be more capable of volunteering (Gesthuizen and Scheepers, 2012). Additionally, the country's educational levels have had a significant impact on volunteering rates, particularly where employment opportunities differ substantially between individuals with lower and higher levels of education (Gesthuizen and Scheepers, 2012). It has been argued that higher-status jobs have a positive influence on volunteering, which explains why lower-educated individuals tend to volunteer less frequently than their higher-educated counterparts. In the Western context, higher positions are often bestowed upon individuals who have taken on some responsibilities and roles in volunteering.

On the one hand, while several previous studies have found a positive association between democracy, social trust, and volunteerism (Gesthuizen and Scheepers, 2012; Baer et al., 2017), some studies have found that wealthier countries, on average, report lower levels of volunteerism (Logan et al., 2020). Our findings suggest that the highest levels of volunteering are found in Africa. A possible explanation for this finding could be that higher levels of religiosity, prevalent in most countries on the continent, might enhance the impact of networks and participation in religious organizations, which play essential roles in civic engagement, social support, and other forms of assistance in Africa. Thus, altruism and collective motivations stemming from religiosity might positively influence volunteering in Africa (Storm, 2015; Bennett, 2015). Another possible explanation could be a tendency within communities to rely on mutual aid and cultural norms of collectiveness in resource-limited settings, which extend beyond the effects of religiosity. In particular, in the Global South, the culture is less individualistic, where families and communities share close bonds and norms of reciprocity. Mutual aid is more substantial and collective participation is valued over individual and market-driven forms (Butcher and Einolf, 2017).

Also, the differences in volunteering observed in our study may be attributed to various ethnic and cultural values and heterogeneity. Aydinli et al. (2013) compare prosocial actions, suggesting that helping outgroup members may occur more frequently in rural and less affluent contexts compared to urban and wealthier settings. Nonetheless, Western and affluent countries are more engaged in long-term formal volunteering.

The pooled gender differences in volunteering, while not significant, were noticeable. In many countries, women were less likely to volunteer than men. This trend aligns with findings from analyses of survey data in Africa (Logan et al., 2020) and Europe (Enjolras, 2021). In contrast, North America showed that women participated in direct volunteering activities more than men. In most African and European countries studied, the expected gender differences in volunteering were confirmed, likely due to higher levels of gender inequality, which influence socialization patterns and participation in the public sphere. We found that older people were generally more likely to volunteer in most countries, although this finding was not statistically significant. Our findings indicate that having a secondary or higher educational status is the most critical factor that enables individuals to volunteer. This conclusion is consistent with previous research by Fondling et al. (2023) and Seabe (2014) in the South African context, as well as Han et al. (2023) in the USA. Additionally, findings from combined analyses of African countries (Logan et al., 2020) and European countries (Gesthuizen and Scheepers, 2012; Enjolras, 2021) also found that individuals with higher education levels are more likely to volunteer. This could be explained by the fact that highly educated individuals have a greater awareness of societal issues and increased self-confidence to volunteer. Education also equips people with knowledge, understanding, and empathy for the problems surrounding them, stemming from their exposure to and interest in current events (Gesthuizen and Scheepers, 2012).

### Strengths and limitations of the study and future research

We pooled and investigated the associations of individual age, gender and level of education using multiple nationally representative datasets and analyzed the data in each country in a unified manner. Compared with a single-country study, our work included 31 countries worldwide and could thus provide a more generalizable estimation. Instead of merging data from all countries and conducting a one-stage analysis, we employed meta-analyses, which allowed the effects of age, gender, and education level to vary across countries (Basagaña et al., 2018). This has enabled the generalizability of volunteer work estimates at the population level, rather than smaller studies that may be based on particular population subsets. The sampling methods and the instruments used adhere to the accepted ethical standards recommended for research. Another strength of the study is that, rather than providing an appraisal and summary of volunteering prevalence data, as in Russell (2016), our study has synthesized empirical evidence on the scope and disparities of volunteering to provide global and continental estimates using readily and publicly available observational data. Our study has contributed to the Plan of Action (2020b) recommendations, advocating for leveraging freely available data sources to analyse volunteerism. Thus, it provides findings showing which groups are more likely to volunteer, which is necessary for optimal interventions.

The limitation of the study is that we have conducted a secondary analysis of data already collected in each country. We had no control over the data collection and management procedures. Additionally, differing survey frames and instruments across contexts may impact the analysis results. Specifically, the difficulty of constructing representative sampling frames in the Global South may influence reliability and validity issues (Russell, 2016). Apart from variables related to civic participation and some variables on reasons for volunteering, the WVS surveys are not as comprehensive in capturing other aspects of volunteerism, such as volunteer empowerment and life satisfaction, which limits their use in providing an in-depth understanding of volunteerism (Salamon et al., 2018). Moreover, self-reported volunteering work activities may be subject to recall and social desirability biases, which could result in overreporting or underreporting certain aspects of participants' experiences. This could introduce biases in data collection concerning volunteering and may have led to inaccurate estimates.

A significant limitation of our study is that it utilized data specific to the World Values Survey in various contexts worldwide, often without substantial adaptation. A case in point was when Russell (2016) obtained significantly different volunteering rates in South Africa, depending on the data source: the Charities Aid Foundation's (CAF) World Giving Index, the International Labour Organization's Manual Volunteering Activity Survey, or Social Surveys in Africa. Suppose the analysis used a different survey, such as the Time Use Survey, which measured volunteering activities with a 24 h recall. The issue of differing methodologies, ranging from the simplistic elicitation of volunteering in any groups, clubs, or organizations to a listing of activities or consideration of volunteering activities within a fixed period window, such as the past 24 h or 12 months, limits proper and robust between-country comparisons. Thus, definitions, contexts, and local adoptions should consider universally agreed-upon measurements and methodologies of volunteering work. Also, the data we analyzed is based on formal volunteering through organizations and associations. Salamon et al. (2018) noted that 70% of global volunteer activity occurs through direct, person-to-person engagement. Thus, our findings in this study could have underestimated the scale of volunteering.

We could have chosen to perform an individual participant data (IPD) analysis with a multi-level approach that accommodates data at both the individual and country levels. Reporting volunteer levels at an aggregate country level is crucial for identifying more receptive groups to volunteer, thus informing policy interventions. Our analysis has provided countries and relevant stakeholders with pooled data on volunteer work, allowing them to assess the scope and scale of volunteer contributions toward the 2,030 Sustainable Development Goals (SDGs). Moreover, several studies have demonstrated that both approaches to meta-analysis yield highly comparable results. Additionally, we acknowledge that using country-level summaries for comparison and synthesis may have obscured intra-country variations in volunteering, which could be significant. For example, country-level summaries may mask intra-country variations, such as rural-urban and provincial disparities, as seen in Fondling et al. (2023), Yimer (2020), and Gramatki and Watt (2020). Furthermore, we could have analyzed differentials in volunteering by other known determinants, such as religious beliefs and practices (for example, the importance of God in their lives and regular church attendance and volunteering), which have consistently been found to be positively associated with volunteering at the individual level (Storm, 2015; Bennett, 2015; Damian, 2018).

Our study has highlighted the methodological and data coverage deficiencies of using cross-sectional surveys to measure volunteerism across all aspects. Due to the limitations inherent in cross-sectional studies, several researchers (e.g., Gesthuizen and Scheepers, 2012; Logan et al., 2020; Enjolras, 2021) have supplemented available survey data with external information in their analyses. We recommend the implementation of stand-alone volunteer household surveys, as was the case with the Time Well Spent national survey on the volunteer experience in the United Kingdom (McGarvey et al., 2019) or specialized and dedicated volunteer survey modules embedded in household health surveys, for example using as the traditional Labor Force Surveys to gather information on a variety of aspects regarding volunteering as outlined in the ILO manual (International Labour Organization, 2021). However, even if a detailed and comprehensive harmonized volunteer measurement tool becomes available, it will still need to be adapted to appropriately measure volunteering within the local contexts of volunteering behaviors (Russell, 2016). In this way, the toll will measure the contribution of volunteering toward socio-economic development and the achievement of the Sustainable Development Goals (SDGs) in countries.

### Conclusions

Our study analyzed data from 37 World Values Survey datasets, which are nationally representative samples from 31 countries. This analysis offers a comprehensive perspective on global volunteering trends, highlighting the cultural, social, and economic factors that influence participation. The findings offer policymakers actionable insights for effectively targeting volunteer initiatives. We have also demonstrated that existing data sources are adequate for measuring and reporting on volunteer work. This is particularly critical given the scarcity of alternative sources that provide high-quality data on volunteer work in most countries. Our analysis indicates that existing data yields valuable insights into the scope and factors influencing volunteering. Our findings reveal that volunteering rates vary considerably across different countries. Education plays a significant role in an individual's likelihood to volunteer. This information could greatly assist policymakers and nonprofit organizations as they promote, plan, and allocate resources for volunteer initiatives.

## Data Availability

Publicly available datasets were analyzed in this study. This data can be found here: https://www.worldvaluessurvey.org/WVSContents.jsp.
